# Effect of Composition, Interface, and Deposition Sequence on Electrical Properties of Nanolayered Ta_2_O_5_-Al_2_O_3_ Films Grown on Silicon by Atomic Layer Deposition

**DOI:** 10.1186/s11671-019-2907-0

**Published:** 2019-03-04

**Authors:** Junpeng Li, Jianzhuo Wu, Junqing Liu, Jiaming Sun

**Affiliations:** Research Center for Photonics and Electronics Materials, School of Materials Science and Engineering and National Institute for Advanced Materials, Nankai University, Tongyan Road 38, Tianjin, 300350 China

**Keywords:** Ta_2_O_5_, Nanolayered films, Electrical property, Atomic layer deposition, Post-annealing

## Abstract

Nanolayered Ta_2_O_5_-Al_2_O_3_ composite films were grown on *n*-type silicon by atomic layer deposition (ALD) within the overlapped ALD window of 220–270 °C. Moreover, post-annealing treatment was carried out to eliminate defects and improve film quality. Nanolayered Ta_2_O_5_-Al_2_O_3_ composite films remain amorphous after 700 °C annealing. The effects of composition, interface, and deposition sequence on electrical properties of Ta_2_O_5_-Al_2_O_3_ composite films were investigated in detail utilizing MIS devices. The results demonstrate that the formation of Ta_2_O_5_-Al_2_O_3_ composite films by mixing Al_2_O_3_ into Ta_2_O_5_ can decrease the leakage current effectively, but it leads to the decrease of the dielectric constant and the enhancement of the hysteresis effect. The interfaces in composite films are not conducive to prevent the leakage current. The deposition sequence of Si/(Al_2_O_3_/Ta_2_O_5_)_n_, Al_2_O_3_ as the first covering layer, reduces the leakage current and the hysteresis effect effectively. Therefore, the electrical properties of Ta_2_O_5_-Al_2_O_3_ composite films could be regulated by adjusting components and structures via ALD to acquire relatively great dielectric constants and acceptable leakage currents.

## Background

With the shrinking of the sizes, the limitations of silicon oxide (SiO_2_) gate dielectric for ultra large-scale integration (ULSI) devices have been reached, hence developing new gate dielectrics for next generation of microelectronic devices has become an urgent task in semiconductor industry [1]. It is required that the leakage current of new gate dielectrics has to be lower than that of the conventional SiO_2_ under the same equivalent oxide thickness. Therefore, various high-*k* dielectric materials have been recommended to replace SiO_2_ [2, 3].

Recently, alternative metal oxide films have been extensively investigated such as Ta_2_O_5_, Al_2_O_3_, ZrO_2_, HfO_2_, Nb_2_O_5_, and TiO_2_. Among them, tantalum pentoxide (Ta_2_O_5_) has been considered as one of the most promising candidates to replace SiO_2_ due to its relatively high dielectric constant of about 20~60 [4–8]. However, Ta_2_O_5_ has noticeable high-field conductivity and cannot prevent carriers leakage due to its small band gap of 4.4 eV, which means this metal oxide cannot be independently used as a dielectric film. Hence, it is necessary to introduce an excellent insulating material to block leakage current [9]. Al_2_O_3_ is one of the most investigated materials with large band gap (8.7 eV) and high breakdown electric field [10–13]. To optimize the electrical property of Ta_2_O_5_ as gate dielectric, ultrathin Al_2_O_3_ can be mixed into Ta_2_O_5_ thin films for its current-blocking capability [14–16]. This composite structure is believed to provide a high dielectric constant and an acceptable leakage current by controlling the composition and structure [17–23].

As for film deposition methods, atomic layer deposition (ALD) based on saturated self-limiting surface reactions has become an important film deposition technique in the semiconductor industry. It exhibits many advantages over other deposition routes, such as precise thickness control at atomic layer level, high uniformity over a large area, excellent conformity in many complex nanostructures, and controllable film structure and composition [24–28]. Min-Kyu et al. [29] reported the film deposition of Ta_2_O_5_ via thermal and ozone (O_3_) ALD using pentaethoxytantlum as Ta precursor. Hyunchol et al. [30] reported the growth of the ZrO_2_/Ta_2_O_5_ multi-laminate films by ALD and the relation between their dielectric and chemical properties. Partida-Manzanera et al. [4] reported (Ta_2_O_5_)_x_(Al_2_O_3_)_1−x_ thin films deposited by ALD using pentakis(dimethylamino)tantalum as Ta precursor and DI water as oxidizer, and the effects of tantalum doping and annealing on dielectric performance. Nevertheless, the effect of composition, interface, and the deposition sequence in composite thin films on electrical properties of Ta_2_O_5_-Al_2_O_3_ film deposited by ALD still need to be further illustrated.

In this work, we deposited nanolayered Ta_2_O_5_-Al_2_O_3_ composite thin films on *n*-type silicon wafers by ALD technology using pentakis(dimethylamino)tantalum (PDMATa) and trimethylaluminum (TAM) as metal precursors, as well as O_3_ as an oxidizer. Moreover, post-annealing treatments were carried out to eliminate defects and improve film quality [31]. The electrical properties of films were studied utilizing the MIS device with Ta_2_O_5_-Al_2_O_3_ as dielectric layer [32]. The effects of film composition, interface, and the deposition sequence on electrical properties of film were investigated in detail by capacitance-voltage and current-voltage measurement.

## Methods

Nanolayered Ta_2_O_5_-Al_2_O_3_ composite films were grown onto oriented *n*-type silicon wafers using an ALD reactor (MNT Ltd.). Trimethylaluminium was held at room temperature and pentakis(dimethylamino)tantalum was heated to 80 °C. Ozone as an oxidant was generated from oxygen (99.999% purity) by an ozone generator (Newland Ltd.). High purity nitrogen gas (99.999%) was used as the carrying and purging gas. Moreover, the temperature of the reactor chamber and the delivery lines was remained at 230 °C and 120 °C, respectively. All the samples were annealed at 700 °C for 2 h under nitrogen ambient. The Al electrodes on both sides of the samples were deposited by physical vapor deposition. The samples were annealed at 250 °C for 0.5 h to assure reliable ohmic contacts. The samples with varying ratios and varying interface number were prepared by controlling the ALD cycles or sub-layer thickness of Ta_2_O_5_ and Al_2_O_3_.

The thicknesses and refractive indexes of all samples were measured by an ellipsometer. The crystal structure of the Ta_2_O_5_-Al_2_O_3_ films was characterized by glancing angle X-ray diffraction (GAXRD) with Cu Kα radiation. Current-voltage (*I-V*) measurements were carried out by a Keithley 2410 1100 V source measurement unit (Keithley Instruments Inc.) and capacitance-voltage (*C-V*) measurements were carried out by TH2828S LCR meter (Tonghui Electronics). All the measurements were completed at room temperature.

## Results and Discussion

**Fig. 1:**
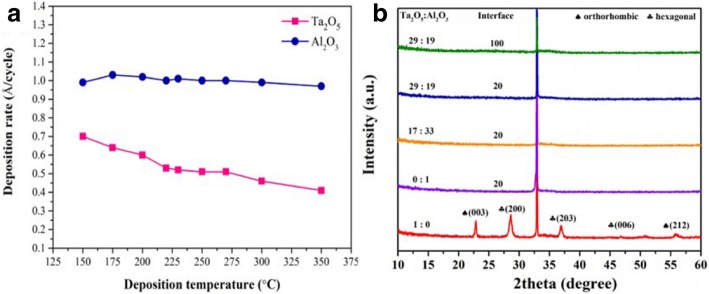
**a** The deposition rate of Ta_2_O_5_ and Al_2_O_5_ as a function of deposition temperatures. **b** The XRD patterns of Ta_2_O_5_, Al_2_O_3_, and Ta_2_O_5_-Al_2_O_3_ composite films annealed at 700 °C

Figure 1a shows the change of deposition rate as a function of deposition temperature. There is an overlap for ALD temperature windows of Ta_2_O_5_ and Al_2_O_3_. Therefore, Ta_2_O_5_-Al_2_O_3_ composite films can be deposited within the temperature range of 220~270 °C, in which it is controllable to grow uniform and high-quality dielectric films by ALD manner. Moreover, the deposition rates of Ta_2_O_5_ and Al_2_O_3_ are constant 0.52 Å/cycle and 1.01 Å cycle in ALD temperature windows, respectively. The deposition rates can be used to design the thickness and component contents of the composite film. Annealing treatment is regarded as a necessary process to eliminate defects and improve film quality [33]. Figure 1b shows the GAXRD patterns of Ta_2_O_5_, Al_2_O_3_, and Ta_2_O_5_-Al_2_O_3_ films annealed at 700 °C. Pure Al_2_O_3_ film remained amorphous state after 700 °C annealing. In the pattern of Ta_2_O_5_, the strong peaks at 22.8° and 56.8° are indexed to the orthorhombic Ta_2_O_5_ (PDF Card 25-0922), and the peaks at 28.5°, 36.9°, and 46.8° are indexed to the hexagonal Ta_2_O_5_ (PDF Card 18-1304). However, no diffraction peak was detected in the pattern of Ta_2_O_5_-Al_2_O_3_ composite films with various composition and interfaces. One possible explanation is that crystallization is inhibited in the ultrathin Ta_2_O_5_ sub-layers. The other is that amorphous Al_2_O_3_ mixed in the composite film increases the crystallization temperature of Ta_2_O_5_ film.

**Fig. 2:**
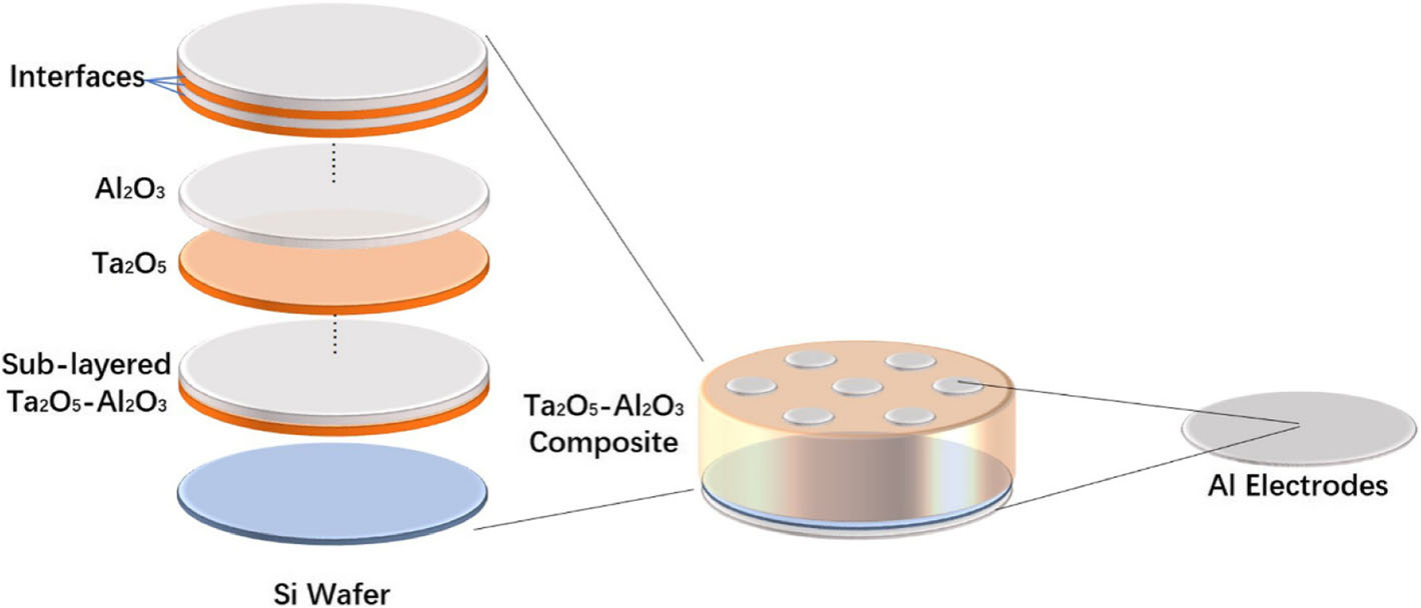
Schematic diagram for Ta_2_O_5_-Al_2_O_3_ composite film and MIS device

Three series of experiments, as shown in Table 1, were carried out to investigate the effects of component ratio, the number of interface, and deposition sequence on electrical properties. The nanolayered Ta_2_O_5_-Al_2_O_3_ composite films have a periodic structure consisted of several sub-layered Ta_2_O_5_-Al_2_O_3_. The electrical properties of composite films were studied utilizing the metal-insulator-semiconductor (MIS) devices, as shown in Fig. 2.

**Table 1:** The experimental design for studying the effects of composition, interface, and deposition sequence on electrical properties

	ALD cycles			Composition (Ta_2_O_5_:Al_2_O_3_)	Interfaces (in film)	Deposition sequence (first layer)
	Ta_2_O_5_	Al_2_O_3_	Major cycle			
I	86	0	10	1:0	0	Ta_2_O_5_
	72	12	10	38:12	20	Ta_2_O_5_
	55	19	10	29:19	20	Ta_2_O_5_
	50	23	10	27:23	20	Ta_2_O_5_
	44	26	10	23:26	20	Ta_2_O_5_
	32	33	10	17:33	20	Ta_2_O_5_
	0	54	10	0:1	0	Al_2_O_3_
II	11	4	50	29:19	100	Al_2_O_3_
	23	8	25	29:19	50	Al_2_O_3_
	43	15	13	29:19	26	Al_2_O_3_
	55	19	10	29:19	20	Al_2_O_3_
	63	22	9	29:19	18	Al_2_O_3_
	80	28	7	29:19	14	Al_2_O_3_
III	72	12	10	38:12	20	Al_2_O_3_
	72	12	10	38:12	20	Ta_2_O_5_

**Fig. 3:**
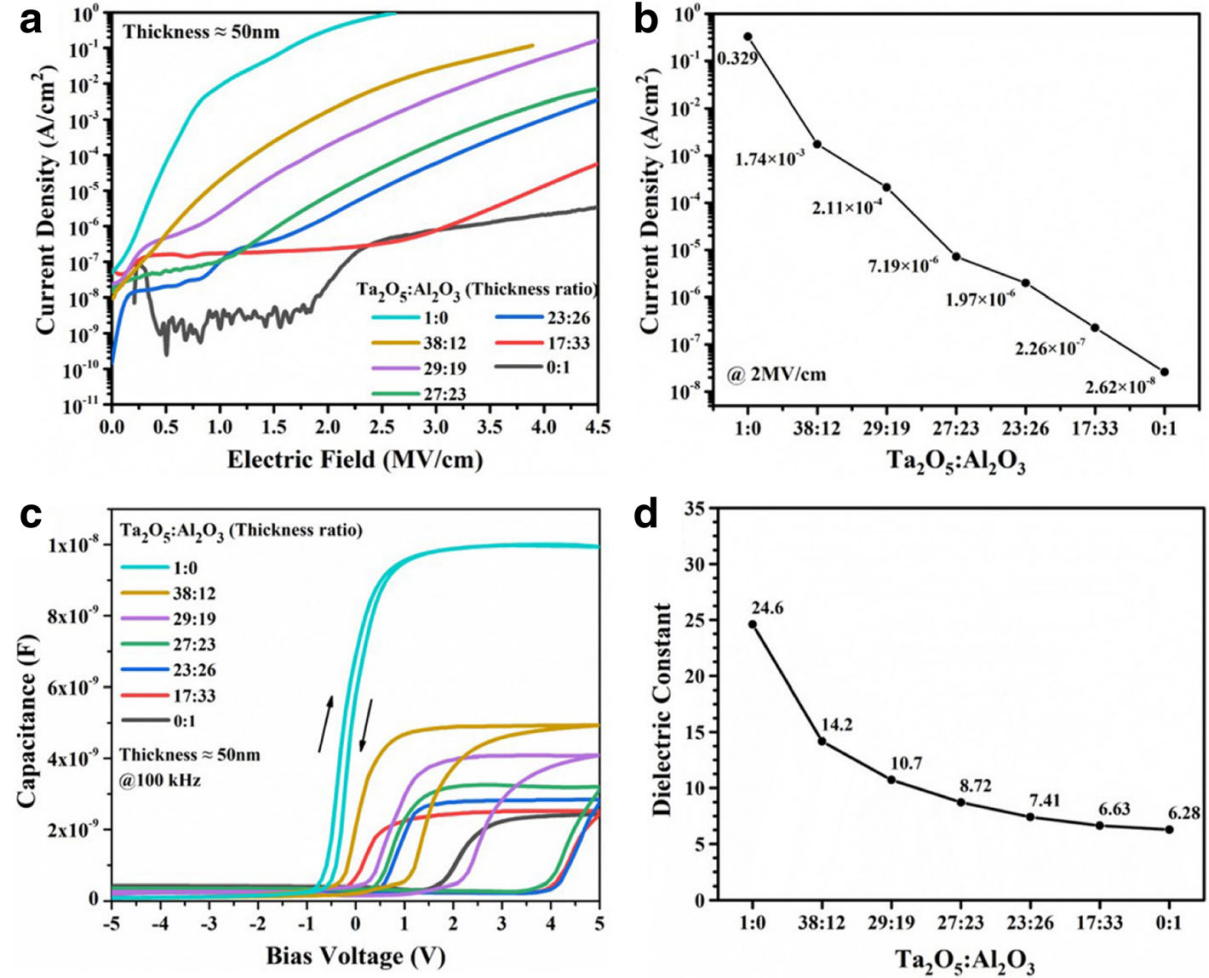
**a** *I-V* behaviors and **b** the leakage current for Ta_2_O_5_-Al_2_O_3_ films with various thickness ratios. **c** *C-V* behaviors of Ta_2_O_5_-Al_2_O_3_ composite films with various ratios of Ta_2_O_5_ to Al_2_O_3_ and **d** dielectric constants calculated from *C-V* results

To study the effect of the component ratio in composite films on the electrical properties, in experiment I, the thickness ratios of Ta_2_O_5_ to Al_2_O_3_ in films varied from 1:0 to 0:1. Figure 3a shows that the curves of current density versus electric field intensity. For pure Al_2_O_3_ film, it is difficult to inject current due to its strong insulativity. For pure Ta_2_O_5_, it shows obvious leakage current and low breakdown field strength. In Fig. 3b, the current density of pure Ta_2_O_5_ (Ta_2_O_5_:Al_2_O_3_ = 1:0) film at 2 MV/cm is 0.329 A/cm^2^ due to high-field conductivity and abundant grain boundary as the leakage paths [34]. Then, the current density decreases correspondingly with decreasing the thickness ratios of Ta_2_O_5_ to Al_2_O_3_ from 1:0 to 0:1, and it finally declines down to 2.62 × 10^−8^ A/cm^2^. The results demonstrate that the mixing Al_2_O_3_ into Ta_2_O_5_ thin film can decrease the leakage current effectively. One reason is Al_2_O_3_ with wide band gap has strong insulativity and can act as a barrier layer to prevent leakage current. The other is that the amorphous phase of composite film blocks leakage current path. To calculate the dielectric constants of Ta_2_O_5_-Al_2_O_3_ composite films, the *C-V* measurement was carried out at 100 kHz at a ramp rate of 100 mV/s, as shown in Fig. 3c. A low capacitance state is a depletion region in the negative voltage range and a high capacitance state is an accumulation region in the positive voltage range for MIS capacitors. The capacitances decrease with reducing the thickness ratio of Ta_2_O_5_ to Al_2_O_3_. Moreover, the *C-V* data of Ta_2_O_5_-Al_2_O_3_ composite films display significant flat band shifts to more positive voltages and additionally significant hysteresis with increasing Al_2_O_3_ content ratios. The positive shifts of flat band voltage can be attributed to the negative charges from trapping of electrons as well as fixed charges at the interface or in the film. Hysteresis effect in *C-V* measurements is normally attributed to charge trapping in the oxide or at the interface, mobile charge, and remnant polarization [35]. In Fig. 3d, the dielectric constant of pure Ta_2_O_5_ (Ta_2_O_5_:Al_2_O_3_ = 0:1) and pure Al_2_O_3_ film was calculated at 24.6 and 6.28, respectively. For Ta_2_O_5_-Al_2_O_3_ composite films, as is expected, the dielectric constants decrease continuously with the increase of Al_2_O_3_ content correspondingly.

**Fig. 4:**
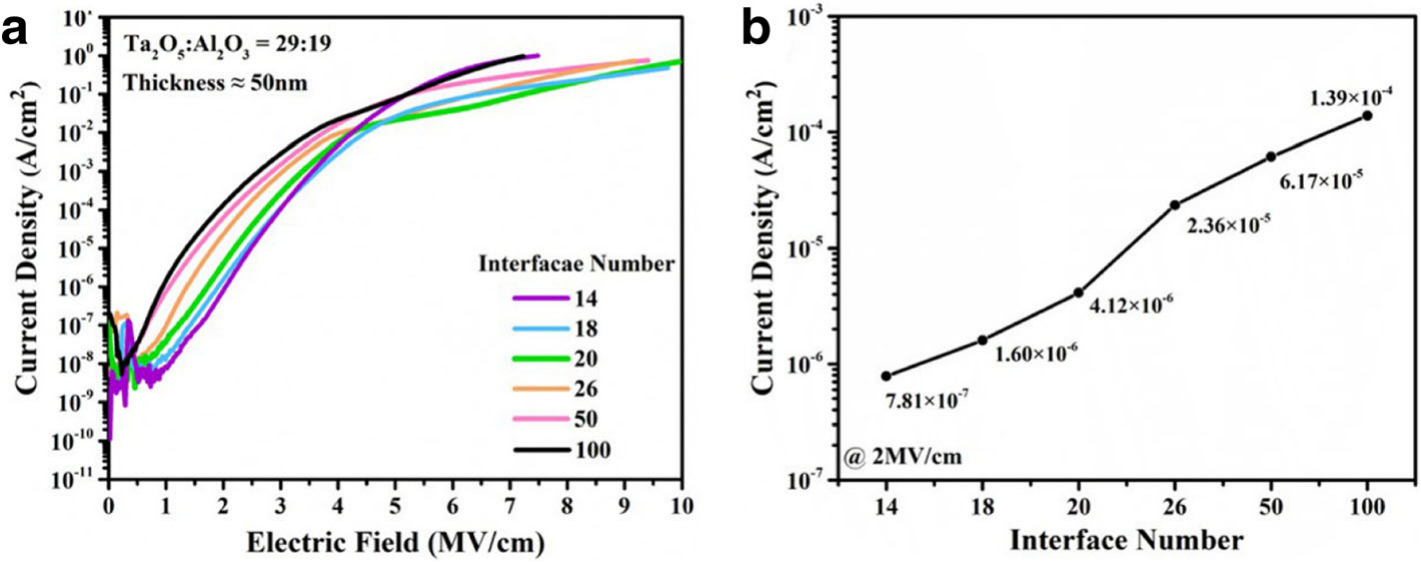
**a** *I-V* behaviors and **b** the change of leakage current for the composite films with various the number of interfaces

**Fig. 5:**
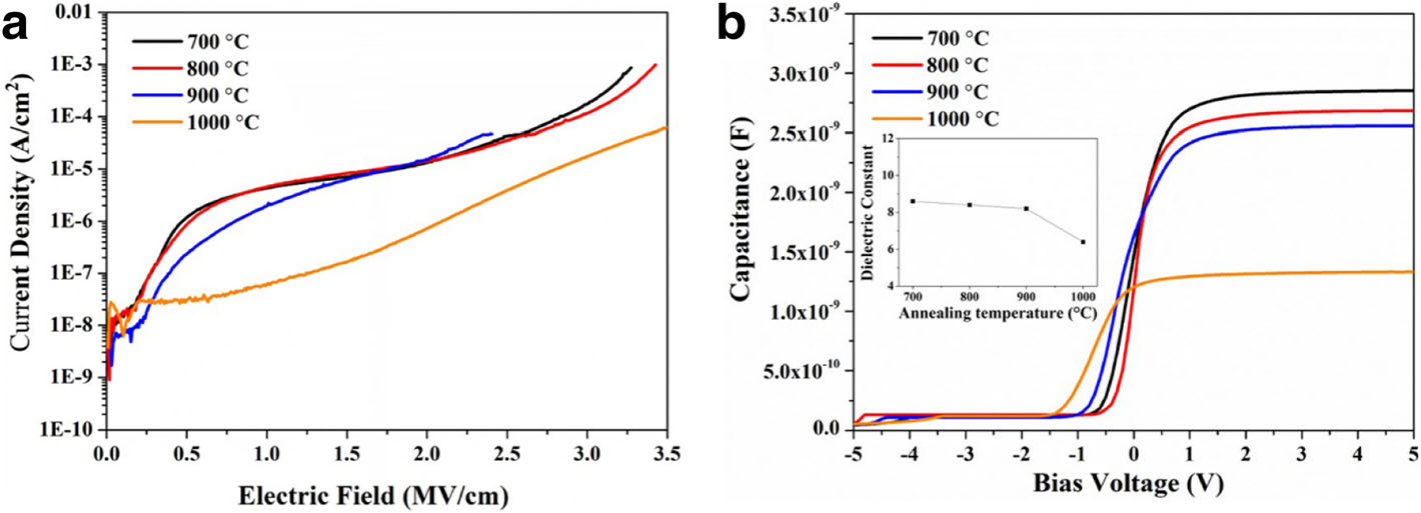
**a** *I-V* behaviors and **b** the *C-V* behaviors of the composite films annealed at 700–1000 °C

To explore the effect of interface in composite films on the electrical properties, in experiment II, the number of the interfaces varied from 14 to 100. Figure 4a shows the leakage current behaviors of Ta_2_O_5_-Al_2_O_3_ composite films with various number of interfaces. It can be found that the interface has smaller effects on leakage current compared to the film component. In Fig. 4b, the current density of Ta_2_O_5_-Al_2_O_3_ composite films is 7.81 × 10^−7^ A/cm^2^ when the number of interfaces is 14, and then it increases continuously with increasing the number of interfaces from 14 to 100 at the electric field of 2 MV/cm. These results demonstrate that interfaces in Ta_2_O_5_-Al_2_O_3_ films are not conducive to prevent the leakage current. These defects trend to generate at interfaces due to the different ionic radius and valence states for Ta^5+^ and Al^3+^. Moreover, more interfaces mean thinner Ta_2_O_5_-Al_2_O_3_ sub-layers in fixed-thickness film. The interface defect density will increase with the reduction of film thickness [36], which may cause an increase of leakage current. In addition, the effect of SiO_2_ interface on the electrical properties of the nanolayered film is relatively minor after 700 °C annealing under N_2_ ambient. Before ALD processes, the native oxide has been removed by an HF last cleaning step immediately before the deposition. The HF step gives rise to a hydrogen-passivated surface, which becomes the initial state for the ALD process. After the film deposition, the samples were annealed at 700 °C under N_2_ ambient. The inert gas can prevent the oxidation of Si and the further growth of SiO_2_ interface. Moreover, the Al_2_O_3_ films are not permeable for oxygen diffusion [37]. Al_2_O_3_ as a barrier layer in nanolayered Ta_2_O_5_-Al_2_O_3_ film can suppress oxygen diffusion toward the interface between Si and nanolayered film. Therefore, the effect of the SiO_2_ interface on the electrical properties of the nanolayered film is limited below 900 °C annealing. However, the SiO_2_ interface has an effect on the electrical properties of nanolayered Ta_2_O_5_-Al_2_O_3_ film when the annealing temperature is above 1000 °C. As shown in Fig. 5, the reduction of leakage current and dielectric constant can be attributed to the growth of the SiO_2_ interface during the annealing processes.

**Fig. 6:**
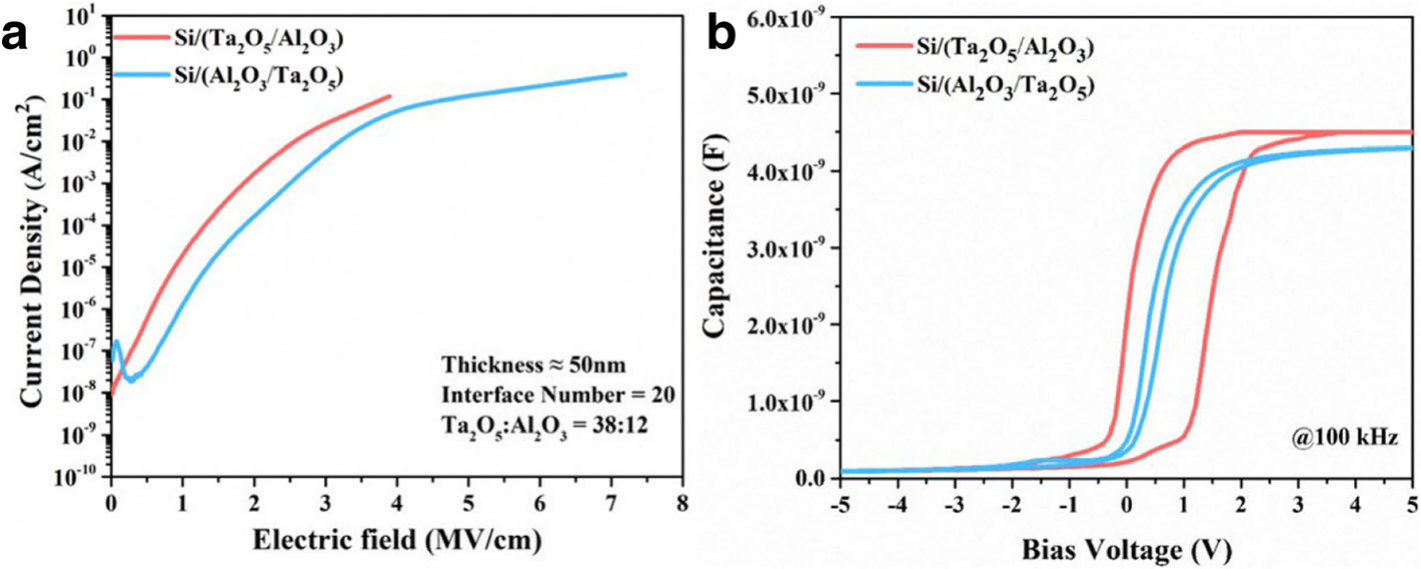
**a** The *I-V* and **b** the *C-V* behaviors of films with the different deposition sequence

The effect of the deposition sequence on the electrical properties was compared in experiment III. The deposition sequence of composite films on silicon was first Ta_2_O_5_ and then Al_2_O_3_, which was defined as Si/(Ta_2_O_5_/Al_2_O_3_)_n_. Otherwise, it was defined as Si/(Al_2_O_3_/Ta_2_O_5_)_n_. Figure 6a, b depicts the leakage current behaviors and the curves of *C-V*. The current density of Si/(Ta_2_O_5_/Al_2_O_3_) film is higher than that of Si/(Al_2_O_3_/Ta_2_O_5_) film at the electric field of 4 MV/cm, and the breakdown field of Si/(Ta_2_O_5_/Al_2_O_3_) film is obviously weaker than that of Si/(Al_2_O_3_/Ta_2_O_5_) film. In addition, the hysteresis of the *C-V* curve for Si/(Ta_2_O_5_/Al_2_O_3_) film is obviously greater. It is reported that Al_2_O_3_ thin film has a low interface trap density [38, 39] and can improve interfacial properties [22]. It can be seen that there are lesser defects at the Si/Al_2_O_3_ interface compared to the Si/Ta_2_O_5_ interface. Moreover, the Al_2_O_3_ films are not permeable for oxygen diffusion. It can act as a barrier layer to cover Si in order to prevent the diffusion of oxygen in film toward Si/Al_2_O_3_ interface.

The above results illustrate that film composition, structure, and interface state density act as the key factors to affect the electrical properties. A compromise property was obtained by mixing Al_2_O_3_ into Ta_2_O_5_ film. The increase of film crystallinity can not only increase the dielectric constant, but also increase the leakage current due to abundant grain boundary as a leakage path. Moreover, high interface state density should be avoided for the laminated or doped film on account of the negative influence on leakage current. Therefore, the amorphous dielectric film with high dielectric constant, relatively large band gap energy, and low interface state density may be a promising gate dielectric to replace SiO_2_. In addition, deposition technology also as a key factor has an important effect on electrical properties of gate dielectric.

## Conclusions

Nanolayered Ta_2_O_5_-Al_2_O_3_ composite films were grown on *n*-type silicon by ALD. The overlapped temperature window for Ta_2_O_5_ and Al_2_O_3_ is 220~270 °C using pentakis(dimethylamino)tantalum as the Ta precursor and O_3_ as the oxidant. Nanolayered Ta_2_O_5_-Al_2_O_3_ composite films remain amorphous after annealing treatment at 700 °C. The formation of Ta_2_O_5_-Al_2_O_3_ composite films by introducing Al_2_O_3_ into Ta_2_O_5_ can decrease the leakage current effectively due to the excellent insulator for amorphous Al_2_O_3_, but lead to the decrease of the dielectric constant. Moreover, the interfaces in composite films are not conducive to prevent the leakage current. In addition, the deposition sequence of Si/(Al_2_O_3_/Ta_2_O_5_)_n_, Al_2_O_3_ as the first covering layer, reduces effectively the leakage current and the hysteresis effect due to its thermostability and barrier effect. Therefore, the electrical properties of Ta_2_O_5_-Al_2_O_3_ composite films could be regulated by adjusting components and structures via ALD to acquire relatively great dielectric constants and acceptable leakage currents.

## Abbreviations

ALD: Atomic layer deposition
*C-V*: Capacitance-voltage
GAXRD: Glancing angle X-ray diffraction
*I-V*: Current-voltage
O_3_: Ozone
PDMATa: Pentakis(dimethylamino)tantalum
TAM: Trimethylaluminum
ULSI: Ultra large-scale integration
